# Examining Crosstalk among Transforming Growth Factor β, Bone Morphogenetic Protein, and Wnt Pathways*

**DOI:** 10.1074/jbc.M116.759654

**Published:** 2016-11-28

**Authors:** Adam D. Coster, Curtis A. Thorne, Lani F. Wu, Steven J. Altschuler

**Affiliations:** From the ‡ Green Center for Systems Biology, University of Texas Southwestern Medical Center, Dallas, Texas 75390 and; the § Department of Pharmaceutical Chemistry, University of California, San Francisco, California 94158

**Keywords:** bone morphogenetic protein (BMP), imaging, signal transduction, transcription factor, transforming growth factor β (TGF-β), Wnt signaling

## Abstract

The integration of morphogenic signals by cells is not well understood. A growing body of literature suggests increasingly complex coupling among classically defined pathways. Given this apparent complexity, it is difficult to predict where, when, or even whether crosstalk occurs. Here, we investigated pairs of morphogenic pathways, previously reported to have multiple points of crosstalk, which either do not share (TGFβ and Wnt/β-catenin) or share (TGFβ and bone morphogenetic protein (BMP)) core signaling components. Crosstalk was measured by the ability of one morphogenic pathway to cross-activate core transcription factors and/or target genes of another morphogenic pathway. In contrast to previous studies, we found a surprising absence of crosstalk between TGFβ and Wnt/β-catenin. Further, we did not observe expected cross-pathway inhibition in between TGFβ and BMP, despite the fact that both use (or could compete) for the shared component SMAD4. Critical to our assays was a separation of timescales, which helped separate crosstalk due to initial signal transduction from subsequent post-transcriptional feedback events. Our study revealed fewer (and different) inter-morphogenic pathway crosstalk connections than expected; even pathways that share components can be insulated from one another.

## Introduction

Morphogenic signals provide extracellular information needed for cells to make decisions during development and differentiation (1, 2). It is not fully understood at which level of processing cells decode combinations of extracellular signals. In principle, cells could integrate morphogenic information at the membrane, during initial cytoplasmic processing, through nuclear transcriptional regulation, or even via post-transcriptional regulation of the input signaling pathways. Current models of signaling crosstalk often fail to (or cannot) distinguish among these processes, making it difficult to predict which inter-pathway connections exist for any given time or experimental context, and which ones are likely to be intrinsic to these pathways or farther downstream (3). There are multiple reports of crosstalk; however, the experimental conditions are widely varied and often use long time lags combined with gene overexpression or silencing/deletion studies. This has led to an understanding that crosstalk is rampant. However, this interpretation is in opposition to the view that there is an underlying simplicity to classical morphogenic signal transduction; minimal cytoplasmic crosstalk prior to transcriptional regulation helps transmit clear and specific signals to elicit irreversible decisions (3, 4).

Here, we examine the presence or absence of morphogenic crosstalk in a specific system, where crosstalk was measured by the ability of one morphogenic pathway to cross-activate core transcription factors and/or target genes of another morphogenic pathway. In this study, we focused on crosstalk between the well studied TGFβ, Wnt/β-catenin, and BMP^4^ pathways for a number of reasons. First, there are numerous claims of crosstalk between these pathways. Many putative interactions have been reported (5–14) for the classical TGFβ and Wnt/β-catenin pathways, although they do not share common signaling components (5–13). For the paralogous TGFβ and BMP pathways, competition for limited quantities of a core signaling component (Smad4) has been suggested as a source of inhibitory crosstalk (15–17). Second, these pathways are deeply conserved across metazoans, essential to development, frequently coordinate cell fate decisions within tissue compartments, and have well established input stimuli (16, 18–29). Third, these pathways co-regulate multiple target genes at the level of transcription, and provide well defined output readouts of translocated transcription factors and transcribed target genes (6, 14, 22, 30–35). Together, the pairs of TGFβ and Wnt/β-catenin and TGFβ and BMP pathways, which do not and do (respectively) share common conserved signaling components, provide contrasting settings of broad biological relevance in which to investigate when and where crosstalk occurs. Because past reports of crosstalk were largely derived from varying experimental systems and time points, we sought a systematic approach to search for evidence of signaling crosstalk by monitoring canonical pathway readouts to combinatorial signaling inputs. We first stimulated cells with single ligand inputs or combinations of ligand inputs. To distinguish levels of crosstalk, we monitored pathway-specific responses based on nuclear translocation of transcription factors as well as pathway-specific mRNA transcriptional levels. We additionally monitored relatively early time points (1–2 h) as well as later time points (18 h) to help separate crosstalk due to initial processes of signal transduction from subsequent transcription and transcriptional feedback events. Our studies revealed a relative absence of signaling crosstalk between the TGFβ and Wnt/β-catenin pathways at early time points; signaling crosstalk was only observed at later time points (18 h), likely due to transcriptional response. Further, despite sharing a core signaling component, SMAD4, we found that the TGFβ and BMP pathways did not display an expected mutual cross-pathway inhibition at early time points; rather, we identified only the presence of activating crosstalk from TGFβ to BMP and no crosstalk from BMP to TGFβ. Our studies suggest that crosstalk is sparser than expected from current studies, and highlight the difficulty of inferring signaling integration based on physical interactions of components alone.

## Results

### 

#### 

##### Quantitation of Morphogenic Signaling Responses

**FIGURE 1.**
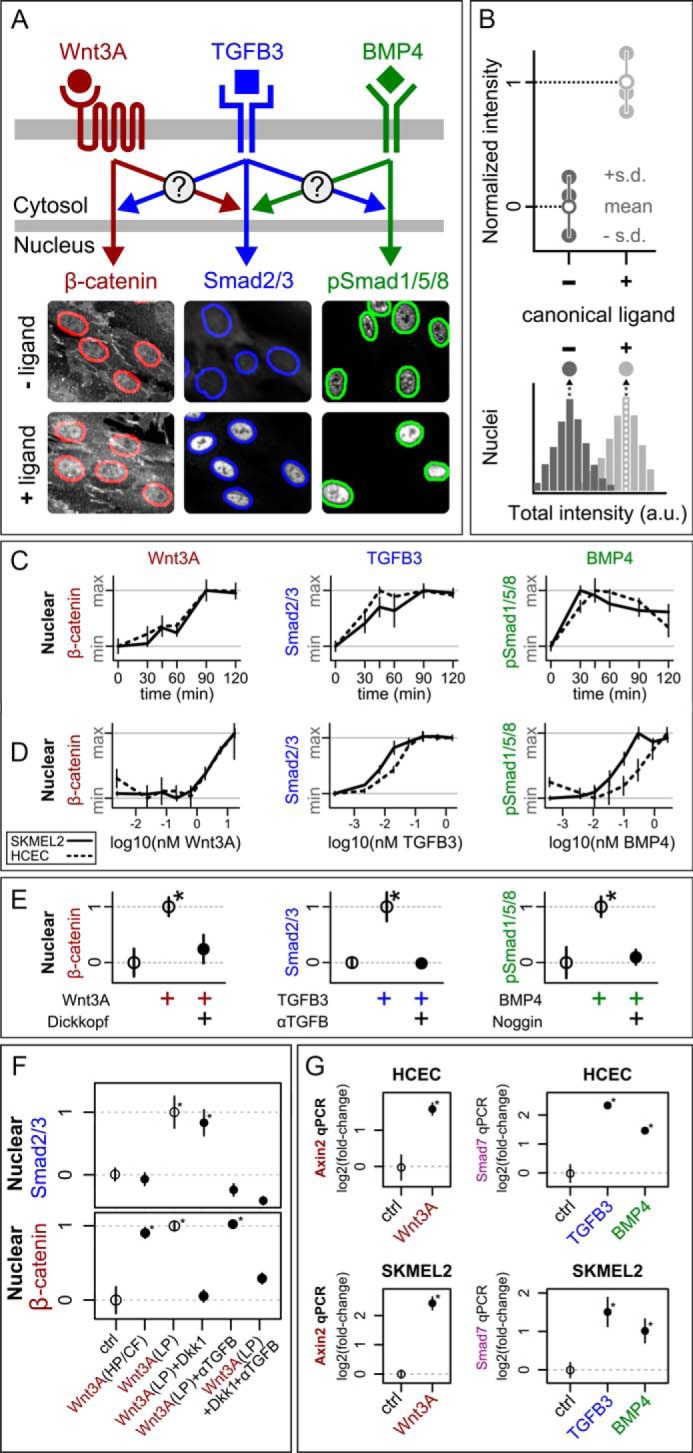
**Establishment of our system for investigating crosstalk.** *A*, overview of experimental approach. Cells were treated with combinations of purified ligands, and nuclear transcription factor responses were measured by single-cell immunofluorescence imaging. Sample images of HCECs after 2 h with or without ligand show that Wnt3A globally increases β-catenin levels, TGFβ3 causes bulk nuclear translocation of Smad2/3, and BMP4 increases nuclear phospho-Smad1/5/8. Nuclei are outlined using the same segmentation approach as in all experiments (see “Experimental Procedures”). *B*, graphic of image-based nuclear transcription factor quantification. Total nuclear intensity was measured for each cell (see “Experimental Procedures”), and the population medians of these values (*filled circles*) from each distribution (*bottom*) were obtained for each of three replicate experiments. The means and standard deviations (S.D.) of these median values were then normalized so that the control mean was 0 and the canonical ligand-only mean was 1 (*top*, *open circles*). *C* and *D*, time-course (*C*) and dose-response curves (*D*) at 1 h for SKMEL2 (*solid lines*) and HCEC (*dashed lines*) in response to Wnt3A (*red*), TGFβ3 (*blue*), or BMP4 (*green*). Values were measured as in *B* and normalized to have the same minimum (*min*) and maximum (*max*) values, *n* = 3 per time point. Concentrations were as follows: Wnt3A (4.8 nm), TGFβ3 (180 pm), and BMP4 (2.6 nm). *E*, the input/output relationships can be blocked by co-treatment with specific antagonists. *Left*, Dickkopf-1 (38 nm) blocks Wnt3A (4.8 nm) → β-catenin in SKMEL2s (2 h). *Middle*, a pan-TGFβ-blocking antibody (αTGFβ, 5 μg/ml) blocks TGFβ3 (450 pm) → Smad2/3 in HCECs (2 h). *Right*, Noggin (4.3 nm) blocks BMP4 (1.9 nm) → pSmad1/5/8 in SKMEL2s (1.5 h). *F*, low-purity Wnt3A (used only in this panel) causes dose-dependent accumulation of Smad2/3. The Wnt → Smad2/3 response is likely due to trace contamination by TGFβ ligands. This response is completely blocked by a pan-TGFβ-blocking antibody (αTGFβ, 5 μg/ml) but not blocked by Wnt antagonist (Dkk1, 38 nm) or observed for high-purity/carrier-free (*HP/CF*) Wnt ligands. Concentrations were as follows: Wnt3A (4.8 nm HP/CF, 4 nm low purity (*LP*)). *ctrl*, control. *G*, cells show transcriptional changes to canonical ligands after 2-h treatments. Concentrations were as follows: Wnt3A (4.8 nm), TGFβ3 (450 pm), and BMP4 (1.9 nm). *Open circles* show reference values used for scaling. *n* = 3 for all points, * indicates *p* value <0.05 compared with control (two-sided *t* test).

Mechanisms of receptor activation and signal transduction for TGFβ, BMP, and Wnt pathways are not fully understood. However, activation of each pathway results in nuclear translocation of specific transcription factors or co-factors. To achieve quantitative readouts of signal transduction, we monitored two of the earliest cellular phenotypes: the nuclear localization of canonical transcription factors for each signaling pathway via microscopy, and subsequent levels of transcription via quantitative PCR (qPCR). We inferred crosstalk by measuring how stimulation of one pathway affects either the nuclear localization or the transcription factor response of another (Fig. 1*A*). We used immunofluorescence microscopy to quantify shifts in nuclear localization of transcription factors due to ligand treatment (Fig. 1*B*).

To study inter-pathway crosstalk prior to transcriptional feedback, we chose time scales that provide maximal transcription factor nuclear localization for each pathway (1 and 2 h, Fig. 1*C*). Second, to enhance the possibility of cross-pathway interaction, we chose input ligand concentrations that yielded strong (between half-maximal to maximal) responses (see example dose-response curves in Fig. 1*D*; concentrations for each experiment are indicated in the figure legends). Third, to investigate potential cell type differences, we performed our assays across two pairs of pathway-responsive cell lines. The first pair consists of human colonic epithelial cells (HCECs (36)) and rat small intestinal epithelial cells (IEC6 (37)), chosen due to the well established importance of TGFβ, BMP, and Wnt signaling in the mammalian gut and the non-transformed state of these lines (38). The second cell line pair consists of two metastatic melanoma lines (SKMEL2 and MALME3M (39)), chosen by screening a panel of cancer lines for responsiveness to the studied pathways and for experimental reproducibility (see “Experimental Procedures”). For clarity of exposition, we show data for HCECs and SKMEL2 and report differences, when observed, for the other cell line models. Fourth, we chose purified recombinant TGFβ3, Wnt3A, and BMP4 as pathway inputs, and chose transcription factors Smad2/3, β-catenin, and phospho-Smad1/5/8 (pSmad1/5/8) as prototypical outputs of classical TGFβ3, Wnt3A, and BMP4 signaling, respectively (17, 40) (Fig. 1, *C–E*). We verified that each input/output response pair could be blocked with a high-specificity antagonist (Fig. 1*E*). Importantly, we note that the commonly used recombinant Wnt3A (R&D Systems 5036-WN, 37 kDa, 75%) and Wnt5A (R&D Systems 645-WN and 645-WN/CF, 38 kDa, 80%) can activate Smad2/3, which is likely an artifact due to trace contamination by TGFβ ligands (Fig. 1*F*); thus, we used the higher-purity Wnt3A (R&D Systems 5036-WNP/CF), which did not activate Smad2/3. Finally, we verified with qPCR that pathway responses to our ligands were sufficient to induce significant changes to downstream transcription (Fig. 1*G*).

##### TGFβ3 and Wnt3A Are Insulated during Signal Transduction

**FIGURE 2.**
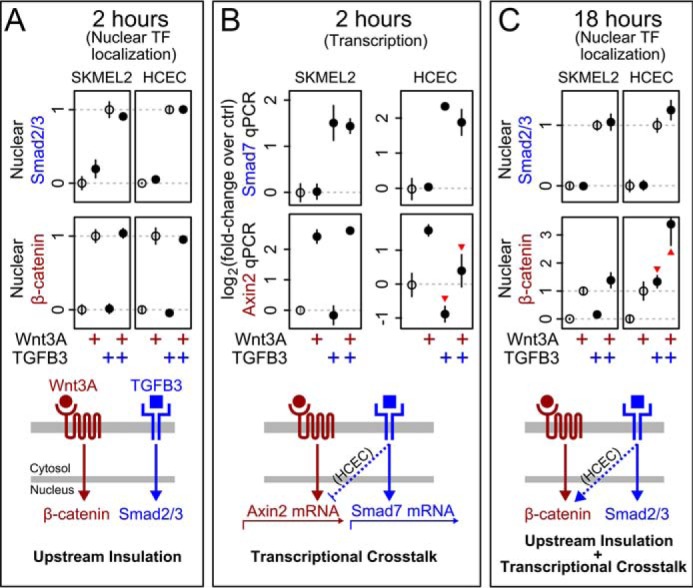
**Wnt3A and TGFβ3 are insulated during signaling but show cell type-dependent transcriptional crosstalk.** *A*, at 2 h, Wnt3A and TGFβ3 show little to no cross-pathway modulation of nuclear transcription factor (*Nuclear TF*) accumulation in HCECs or SKMEL2s. Ligand concentrations were as follows: Wnt3A (2.4 nm) and TGFβ3 (9 pm). *B*, *red arrowheads* indicate HCEC-specific 2-fold reduction of Axin2 expression caused by TGFβ3 at the same 2-h time point (measured by qPCR, see “Experimental Procedures”). *C*, by 18 h, dramatic HCEC-specific activation of β-catenin by TGFβ3 is observed (*arrowheads*). *A* and *C*, data as in Fig. 1*B*, with *open circles* showing reference values used for scaling. *B* and *C*, ligand concentrations were as follows: Wnt3A (4.8 nm) and TGFβ3 (450 pm). *A–C*, *n* = 3 for all points. The no-treatment and canonical ligand-only treatment are significantly different in all cases (*p* value <0.05 in two-sided *t* test).

We first tested for evidence of signaling crosstalk induced by TGFβ3 and Wnt3A. We measured the responses at 2 h of either Smad2/3 or β-catenin to each ligand or combination of ligands. We found that Smad2/3 showed little or no response to Wnt3A input and, similarly, β-catenin showed little or no response to TGFβ3 input (Fig. 2*A*). This was true across cell lines tested at both 1 h and 2 h. Thus, we did not observe crosstalk induced by TGFβ3 and Wnt3A as measured by nuclear accumulation of canonical pathway transcription factors at these early response times.

We then tested for evidence of transcriptional crosstalk induced by TGFβ3 and Wnt3A. We measured expression of the downstream target genes *Smad7* (for TGFβ3) (41) and *Axin2* (for Wnt3A) (3). We found in HCECs that TGFβ3 strongly suppressed Axin2 mRNA expression (Fig. 2*B*, *red arrowheads*), although nuclear levels of β-catenin were unaffected at the same 2-h time point (compare with Fig. 2*A*). This transcriptional crosstalk was maintained in HCECs for at least 6 h (data not shown).

We wondered whether the HCEC-specific suppression of Axin2 mRNA expression induced by TGFβ3 would result in long-term increases of β-catenin protein, as Axin2 protein is a negative regulator of Wnt/β-catenin signaling (42, 43). We repeated the experiment at 18 h to allow time for observing the effects of transcriptional feedback. Consistent with our expression data, HCECs displayed large cross-pathway modulation at this later time point (Fig. 2*C*, *arrowheads*). Indeed, treatment of HCECs with TGFβ3 alone for 18 h was sufficient to increase nuclear β-catenin, and co-treatment with Wnt3A yielded an even larger effect. Cross-pathway modulation at 18 h did not occur in the other cell lines. Thus, signaling crosstalk between TGFβ3 and Wnt3A/β-catenin is a (cell line-dependent) consequence of long-term transcriptional feedback.

##### BMP4 and TGFβ3 Do Not Exhibit Inhibitory Crosstalk

**FIGURE 3.**
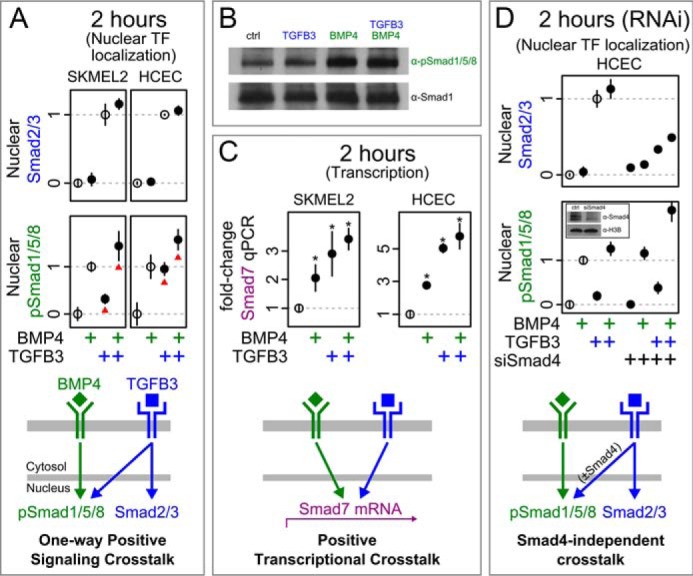
**TGFβ3 and BMP4 do not inhibit one another during signal transduction.** *A*, Smad2/3 (*blue*) does not respond to BMP4, but there is activation of pSmad1/5/8 (*green*) by TGFβ3 (*red arrowheads*). *Nuclear TF*, nuclear transcription factor. *B*, Western blotting confirms the lack of cross-pathway inhibition in SKMEL2 cells after 2-h treatments. Ligand concentrations were as follows: TGFβ3 (450 pm) and BMP4 (950 pm). *C*, TGFβ3 and BMP4 both cause Smad7 expression. * indicates significant departure from the no-treatment case (*p* value <0.05, two-sided *t* test). *D*, Smad4 RNAi in HCECs changes overall TGFβ3 responsiveness (*blue*), but the cross-pathway interactions remain positive. *Inset*, Western blot after Smad4 RNAi shows reduced protein levels of Smad4 in HCECs. *ctrl*, control; *H3B*, histone H3B. Ligand concentrations were as follows: TGFβ3 (450 pm) and BMP4 (950 pm).

To investigate crosstalk between the paralogous TGFβ3 and BMP4 pathways, we measured responses of downstream transcription factor readouts Smad2/3 and pSmad1/5/8 to combinatorial inputs at 2 h. In all tested cell lines, we found cross-pathway activation of pSmad1/5/8 by TGFβ3, consistent with reports in the literature (14, 44–47). As mentioned previously, it has been proposed that the TGFβ superfamily members might inhibit one another via competition for Smad4 (15–17). Interestingly, co-treatment by both ligands did not decrease the relative nuclear localization of either transcription factor (Fig. 3*A*). We confirmed this absence of inhibitory cross-regulation by Western blotting (Fig. 3*B*) and further found that this absence extended to the transcriptional regulation of *Smad7*, a prototypical readout of both pathways (Fig. 3*C*). Therefore, TGFβ3 and BMP4 do not exhibit inhibitory crosstalk as measured by nuclear accumulation of their prototypical transcription factors.

The absence of cross-pathway inhibition could be due to non-limiting quantities of Smad4 in the cell lines tested. Non-limiting abundance of Smad4 is consistent with previous studies (48–51) but may not be a general property across cell lines. We therefore reduced Smad4 levels by siRNA before repeating our test for cross-pathway inhibition between TGFβ3 and BMP4. Although reduction of Smad4 did cause changes to pathway responsiveness, it did not induce a negative interaction between the two pathways (Fig. 3*D*). We therefore found no evidence that TGFβ3 and BMP4 signal transduction pathways negatively regulate one another via competition for Smad4.

## Discussion

Crosstalk between pathways in the larger context of the organism can be expected to occur in many specialized circumstances. A single growth factor can change the biochemical state of a cell, driving differentiation or proliferation and indirectly affecting other pathways by many routes. However, this is generally not what is meant by pathway crosstalk: pathway crosstalk is understood to be a property of the core pathway components themselves and a consequence of interactions that are proximal in space and time. As the results of more and more studies are combined, core signaling pathways are appearing to be increasingly interconnected. It is a challenge to make sense of these complex, static signaling models, which may not describe the operational wiring of any given cell and time. It is, in fact, conceivable that such apparent complexity is hiding a reality of underlying independence (3). For example, complex static signaling networks may be composed of simpler networks that evolve over time (52, 53), in which case only a subset of discovered network connections may be operating for a given biological context and point in time. The concept of dynamic crosstalk is particularly important for developmental pathways, where long time scales may be required for cells to process signals and initiate cellular fate transcriptional programs.

We investigated crosstalk between Wnt3A/β-catenin, TGFβ3, and BMP pathways, as defined by changes in localization of core transcription factors and target gene expression. Our finding that the TGFβ3 and Wnt3A/β-catenin pathways are insulated during early signal transduction stands in apparent contradiction to the body of literature that shows cross-pathway protein-protein interactions (5–13). There are several possible explanations for this discrepancy. First, the bulk of these studies rely on overexpression assays that may generate connections that do not normally exist (54), and/or long-term transcriptional readouts that may conflate direct events during early transduction with the consequences of transcriptional feedback (compare Fig. 2, *A* and *B*, with Fig. 2*C*). Second, our finding that a commonly used recombinant Wnt3A may be contaminated with trace TGFβ ligands leads to uncertainty in the interpretation of previous crosstalk studies using this reagent (see “Experimental Procedures” and Fig. 1*F*). We have not tested multiple lots of commercial Wnt3a, but our results suggest caution when interpreting studies of crosstalk when using lower purity commercial Wnt3a, or any morphogenic ligand for that matter. Third, as our studies with multiple cell lines demonstrate, the TGFβ3 and Wnt3A/β-catenin pathways can show general insulation during upstream signaling while simultaneously showing cell line-dependent transcriptional crosstalk. Finally, even if protein-protein interactions between the TGFβ and Wnt pathways occur, these interactions need not necessarily carry signaling information or alter the process of signal transduction.

We had expected that the TGFβ3 and BMP4 pathways would display cross-pathway inhibition during signal transduction, especially after depletion of Smad4. Instead, we found activating crosstalk from TGFβ3 to the BMP4 pathway and no crosstalk in the other direction. Although we cannot rule out that the absence of cross-pathway inhibition was due to non-limiting Smad4 even after its reduction, the resulting decrease in Smad2/3 activity argues against this. An additional potential explanation could be that the well established nucleocytoplasmic shuttling of Smads (50, 55–57) requires only transient interactions with a shared pool of Smad4. Although the TGFβ superfamily of pathways are some of the best studied and understood cellular signaling networks, many questions remain unresolved regarding downstream signaling dynamics and mechanisms including the stoichiometric and stability requirements for Smad4 interactions (20, 57).

Taken together, our study revealed fewer (and different) inter-morphogenic pathway crosstalk connections than expected from the literature. It is possible that other components or measured phenotypes for the same pathways may reveal different patterns of crosstalk due to the activation of parallel pathways (58), or the use of temporal and other encodings. Indeed, there is evidence that response amplitudes (as measured in this study) carry relatively small amounts of information (41, 59), that TGFβ signaling may also be encoded by pulsatile behaviors (57), and that extracellular Wnt concentrations may be less informative than is widely believed (60). Nevertheless, at least for regulating changes in nuclear transcription factor concentrations and/or target gene expression, our results simplify current models of pathway crosstalk. The relative sparseness of crosstalk connections stands in contrast to the heavily inter-connected growth factor pathways (3, 61). It will be important to determine whether, by separating temporal points of crosstalk, similar simplifications may be found across additional developmental pathways, (*e.g.* Notch and Hedgehog), biological contexts, and information channels.

## Experimental Procedures

### 

#### 

##### Cell Culture

HCECs were a kind gift from Dr. J. Shay (University of Texas Southwestern Medical Center), and the other cell lines were obtained from the American Type Culture Collection. Cell culture was performed under standard culture conditions. In brief, cells were maintained at 37 °C and 5% CO_2_ in RPMI 1640 (Corning Cellgro® 10-040) with 5% FBS (Gemini Bio-Products 100-106, West Sacramento, CA) and antibiotics/antimycotics. We verified that overnight starvation (via the absence of FBS) did not result in qualitatively different study results. For imaging experiments, cells were plated at 2000 cells/well in 384-well glass-bottom plates (Thermo Scientific^TM^Nunc^TM^ 164586). Cells were left to adhere overnight, leading to near confluence, and treated the following day. Although confluency affected signaling, it did not qualitatively change the experimental outcomes (*e.g.* pathways remained insulated).

For treatments, recombinant proteins were diluted into the same medium and added to wells at time = 0. Concentrations (estimated from manufacturer-supplied quantity, purity, and molecular mass) and treatment durations are indicated in the figure legends. Treatments (supplier, product number, approximate molecular mass, approximate purity) are as follows: Wnt3A (high-purity, R&D Systems 5036-WNP/CF, 37 kDa, 90%); Wnt3A (low-purity, R&D Systems 5036-WN, Lot RSK311102B, 37 kDa, 75%); TGFβ3 (Cell Signaling Technology 8425, 22 kDa (dimer), 98%); BMP4 (Cell Signaling Technology 4697, 26 kDa (dimer), 95%); Dickkopf-1 (R&D Systems 5439-DK, 26 kDa, 95%); Noggin (R&D Systems 6057-NG, 23 kDa (monomer), 95%); and αTGFβ blocking antibody (R&D Systems MAB1835).

##### Immunostaining

All solutions were made in PBS (Life Technologies Gibco® 70013). All wash steps were performed three times using 0.1% Tween 20 (Fisher Scientific BP337) in PBS. Antibodies were diluted into 2.5% BSA (Jackson ImmunoResearch Laboratories 001-000, West Grove, PA) After treatment, cells were fixed in 4% paraformaldehyde (Electron Microscopy Sciences 15710, Hatfield, PA) for 10 min, permeabilized with 0.2% Triton X-100 (Sigma-Aldrich 93443) for 10 min, and then washed. Samples were incubated overnight at 4 °C with primary antibodies: β-catenin (1:100 dilution, BD Biosciences 610154), Smad2/3 (1:1000, Cell Signaling Technology 8685), and pSmad1/5/8 (1:100, Cell Signaling Technology 9511). Samples were then washed, stained with 1:1000-diluted secondary antibodies (Alexa Fluor 488/546 αRabbit/αMouse, Life Technologies A11008/A11003) and 2.5 μg/ml Hoechst for 2 h, and washed again. Secondary antibody alone was added to empty wells to serve as references for estimation of uneven illumination.

##### Image Acquisition and Correction

All images were taken using a Nikon® Eclipse Ti-E2000 microscope controlled by NIS-Elements AR V4, with an Andor Zyla sCMOS 11-bit camera, 20× objective lens, and DAPI, FITC, and TRITC filter sets. Images for Fig. 1*E* were taken on an IN Cell Analyzer 6000. All image corrections and analyses were performed using custom MathWorks Matlab® software. Uneven illumination correction and background subtraction were performed as described previously (62). In brief, the detector value (measured by imaging without a light source) was per pixel-subtracted from all images, the shading patterns were estimated per within-well position using uniformly fluorescent reference wells and corrected multiplicatively, and background was estimated per image as the mean of pixel values <0.001 quantile.

##### Nuclear Segmentation, Measurement, and Quality Control

Cellular nuclei were identified in corrected images by simple, automated threshold segmentation. Multiple features were then calculated for each nucleus (including area, total intensity, and coefficient of variation of intensity), for each fluorescence channel. Analysis was restricted to G_1_/_0_ cells, as these were typically the most abundant and less likely to be the result of mis-segmentation. G_1_/_0_ cells were defined as those within ±2 S.D. of the first peak after fitting two Gaussian distributions to the single-cell distribution of total nuclear Hoechst intensity (a proxy for DNA content). G_1_/0 cells were further restricted to those within ±2 median absolute deviations of the median nuclear size as well as the median Hoechst coefficient of variation of intensity (a proxy for texture). The single-cell intensity values of the remaining cells were used for analysis. All observed response distributions for were unimodal, and treatments did not strongly affect distribution shapes (data not shown). Thus, in our subsequent analyses of G_1_/0 cells, we made use of population-level metrics, such as mean or median (Fig. 1*B*).

##### Screening for Pathway-responsive Cell Lines

A subset of ∼25 cell lines from the NCI, National Institutes of Health (NCI-60 Human Tumor Cell Lines Screen) (63) was screened for responsiveness to TGFβ1 (Life Technologies PHG9204) and Wnt3A, and for experimental reliability when using immunofluorescence microscopy. We ranked all cell types by the absolute mean fluorescence intensity change after treatment and then chose the SKMEL2 and MALME3M cell lines because: they were ranked near the top between two independent TGFβ1→Smad2/3 screens, they were ranked near the top for Wnt3A→β-catenin responses, and they satisfied experimental criteria for our image-based studies. These criteria included adherence to glass and plastic imaging surfaces, uniform cell morphology, and good segmentability.

##### Gene Expression

Cells were cultured as above in 96-well plates (BD Falcon^TM^ 353219), with triplicate wells per condition. An Ambion® TaqMan® Cells-to-CT^TM^ (Life Technologies 4391848) kit was used according to the manufacturer's instructions to extract mRNA and create cDNA libraries. qPCR was performed by the University of Texas Southwestern Microarray Core Facility using TaqMan® probes for Axin2 (Hs0061034_m1), Smad7 (Hs00610344_m1), and 18S rRNA (Hs99999901_s1). Triplicate technical replicates were performed per experimental replicate, and the means of these were then used for normalization and statistics. The core facility was blinded to sample identity by obfuscation of sample positions and identifiers. Threshold cycle values were normalized against 18S rRNA values.

##### Western Blotting and RNAi

SDS-PAGE and Western blotting were performed using standard techniques. In brief, cells were grown and treated in 6-well plates, and then lysed with radioimmunoprecipitation assay buffer (50 mm Tris (pH 8.0), 150 mm NaCl, 1% NP-40, 0.5% deoxycholic acid, 0.1% SDS, 0.5 mm EDTA) containing protease and phosphatase inhibitors. The following antibodies were used at 1:1000 dilution: H3B (Cell Signaling Technology 9715), Smad4 (Cell Signaling Technology 9515), Smad1 (Cell Signaling Technology 6944), and pSmad1/5/8 (Cell Signaling Technology 9511). For RNAi, cells were transfected with Dharmacon Smad4 siGENOME SMARTpool (GE Life Sciences M-003902-01) using Lipofectamine® RNAiMAX (Life Technologies 13778075) for 48 h according to the manufacturer's instructions. Transfected cells were then treated as above.

## Author Contributions

A. D. C. and C. A. T. designed and performed the experiments. A. D. C. analyzed the data. A. D. C., C. A. T., L. F. W., and S. J. A. wrote the manuscript. All authors reviewed the final manuscript.

## Footnotes

4 The abbreviations used are:

BMP: bone morphogenetic protein
qPCR: quantitative PCR
HCEC: human colonic epithelial cell
pSmad: phospho-Smad
TRITC: tetramethylrhodamine isothiocyanate.
